# Patterns of Restricted and Repetitive Behaviors in Autism Spectrum Disorders: A Cross-Sectional Video Recording Study. Preliminary Report

**DOI:** 10.3390/brainsci11060678

**Published:** 2021-05-21

**Authors:** Enzo Grossi, Elisa Caminada, Michela Goffredo, Beatrice Vescovo, Tristana Castrignano, Daniele Piscitelli, Giulio Valagussa, Marco Franceschini, Franco Vanzulli

**Affiliations:** 1 Autism Research Unit, “Villa Santa Maria” Foundation, 22038 Tavernerio, Italy; Enzo.Grossi@bracco.com (E.G.); elisa.caminada@villasmaria.org (E.C.); beatrice.vescovo@villasmaria.org (B.V.); tristana.castrignano@villasmaria.org (T.C.); giulio.valagussa@gmail.com (G.V.); franco.vanzulli@villasmaria.org (F.V.); 2 Neurorehabilitation Research Laboratory, Department of Neurological and Rehabilitation Sciences, IRCSS San Raffaele Pisana, 00163 Rome, Italy; marco.franceschini@sanraffaele.it; 3 School of Medicine and Surgery, University of Milano Bicocca, 20126 Milano, Italy; daniele.piscitelli@unimib.it; 4 School of Physical and Occupational Therapy, McGill University, Montreal, QC H3G 1Y5, Canada; 5 Department of Human Sciences and Promotion of the Quality of Life, San Raffaele University, 00163 Rome, Italy

**Keywords:** autism spectrum disorder, repetitive behaviors, classification, real-world data, video recording, motor stereotypies, rehabilitation

## Abstract

Background: Several instruments have been proposed to investigate restricted, repetitive behaviors (RRBs) in individuals with Autism Spectrum Disorder (ASD). Systematic video observations may overcome questionnaire and interview limitations to investigate RRBs. This study aimed to analyze stereotypic patterns through video recordings and to determine the correlation between the number and appearance of RRBs to ASD severity. Methods: Twenty health professionals wearing a body cam recorded 780 specific RRBs during everyday activities of 67 individuals with ASD (mean age: 14.2 ± 3.72 years) for three months. Each stereotypy was classified according to its complexity pattern (i.e., simple or complex) based on body parts and sensory channels involved. Results: The RRBs spectrum for each subject ranged from one to 33 different patterns (mean: 11.6 ± 6.82). Individuals with a lower number of stereotypies shown a lower ASD severity compared to subjects with a higher number of stereotypies (*p* = 0.044). No significant differences were observed between individuals exhibiting simple (*n* = 40) and complex patterns (*n* = 27) of stereotypies on ASD severity, age, sex, and the number of stereotypes. Conclusions: This study represents the first attempt to systematically document expression patterns of RRBs with a data-driven approach. This may provide a better understanding of the pathophysiology and management of RRBs.

## 1. Introduction

Repetitive behaviors have been associated with different disorders such as autism [1]. Their variety is particularly large including motor, sensorial, vocal, and intellective components. These behaviors are inconsistently demonstrated over time, are not always present in the same individual, and can change in quantity, quality, and type. The repetitive use of objects, repetitive activities or ritualism, and repetitive speech are considered repetitive behaviors [2,3]. Numerous studies have addressed behavioral challenges and special interests, e.g., motor stereotypies.

Motor stereotypies denote repetitive, fixed, and purposeless behavioral actions that generally stop with distraction. They involve features such as invariance and repetition [1]. They are not exclusive to humans, being also observed in many animal species [4].

Although motor stereotypies usually are a sign of severe neuropsychiatric conditions such as Autism Spectrum Disorder (ASD), serious sensory deafferentation, intellectual disability, and genetic syndrome [5], they can also be observed in the behavioral repertoire of neurotypical subjects. We refer to both typically developing children as well as adults who, at times, exhibit, for example, the rhythmic swaying of the head and/or the trunk, ritualized behaviors in falling asleep or in frustrating situations [6,7].

Primary simple motor stereotypies (e.g., behavior consisting of one movement: body rocking, tapping, and head-nodding) [8] are reported to occur in roughly 20%–70% of typically developing children, whereas the prevalence of complex motor stereotypies (e.g., behavior consisting of repeated sequences of movements in various body regions: repetitive hand flapping and/or arm and/or, and wiggling movements) is reported in roughly 3%–4% [9,10,11,12,13]. However, complex stereotypies in neurotypical children might be misdiagnosed [11], and prevalence estimates might be compromised. Notably, even though typically developing children show motor stereotypies at an earlier age, they tend to diminish over time, i.e., two years old [10,14]. On the other hand, once stereotypies appear, generally before age three [15], they tend to last for several years, as evidenced by an extensive longitudinal study carried out on children and adolescents with primary complex motor stereotypies. In this study, 98% of the participants continued to exhibit repetitive behaviors up to twenty years old [16].

In children with developmental disabilities, the prevalence of motor stereotypies can be as high as 61% and even higher (88%) in children with ASD [17]. Recently, Melo, Ruano, Jorge, Pinto Ribeiro, Oliveira, Azevedo, and Temudo [3] reported a higher prevalence of motor stereotypies in individuals with ASD and lower Intellective Quotient (IQ), while gender was not associated with its prevalence. Children and adolescents with ASD may also show postural deficits compared to typically developed peers [18].

According to the lexicon of the Diagnostic and Statistical Manual of mental disorders fifth edition (DSM-5) [19], the broad term repetitive behaviors become “Restricted, repetitive and stereotyped patterns of behavior, interests, and activities” (RRBs). Particularly, DSM-5 for the RRB domain is polythetic, i.e., two of four RRBs should be present (reviewed in Burns and Matson [20]). The RRBs range from stereotyped motor movements to atypical reactions to sensory inputs [19]. Turner [1] categorized this broad spectrum of behaviors into two classes: (1) ‘‘lower-level’’ characterized by the repetition of movement including stereotyped movements, repetitive manipulation of objects, and repetitive forms of self-injurious behavior, and (2) ‘‘higher-level’’, which includes object attachments, insistence on sameness, repetitive language, and circumscribed interests. Lower-level behaviors have been found to be associated with lower cognitive abilities, more deficient adaptive skills, and younger chronological age, whereas higher-level behaviors have been shown to be either of no relationship or positive relationships with the same variables [1,3].

Despite their high frequency and strong diagnostic significance within ASD, RRBs have not been fully understood due to their broad spectrum of presentation and pattern complexity [3]. Moreover, Matson et al. [21] suggested that stereotypies could be detected in early life stages as a salient feature of toddlers with autism.

The pathophysiology of RRBs is not fully understood (reviewed in Peter et al. [22]) The disruption of the prefronto-corticobasal ganglia or cortico-striatal thalamo-cortical pathways [23] has been related to stereotypes. The overstimulation of dopaminergic systems has been shown to be associated with the appearance of stereotypies following the intake of levodopa, amphetamine, and cocaine in animal models [24,25]. Motor stereotypies have been elicited in dopamine transporter knock-out mice, suggesting the relation between stereotypies and dopamine pathways [26]. Repetitive behaviors have also been induced, modulating the striatonigral direct circuit in mice models [27]. Imaging studies in subjects with motor stereotypies found lower levels of GABA, supporting the involvement of the anterior cingulate cortex and the striatum in the pathophysiology of motor stereotypies [28]. However, studies performed with different functional and structural procedures did not find a consistent pattern of neuroanatomical characteristics [29], highlighting that physiological and functional disruptions should be investigated than anatomical alterations.

From a clinical perspective, several instruments have been proposed to investigate and assess RRBs. Recently, a panel of experts reviewed twenty-four instruments developed to measure RRBs in subjects with ASD [30]. Several challenges in measuring stereotypies were highlighted, e.g., a variety of clinical presentation in children with ASD, the inter-individual repertoire of stereotypies as well as the prevalence of stereotypies in typically developing children. On the other hand, systematic video observations may overcome questionnaire and interview limitations to investigate stereotypical behaviors [29]. Specifically, standardized video recordings can help depict the intricate pattern of RRBs commonly observed in ASD [31]. Recently, Melo, Ruano, Jorge, Pinto Ribeiro, Oliveira, Azevedo, and Temudo [3] suggested describing and characterizing stereotypies employing direct observation methods, e.g., video recording. A better description and understanding of the variety of repetitive behaviors in individuals with ASD would increase the current knowledge of their pathophysiology and the development of better and more appropriate treatment interventions. We hypothesized that using video recordings in a natural environment during everyday activities would allow classifying RRBs systematically. We also hypothesized that ASD severity might be related to the number and quality of RRBs displayed. To test these hypotheses, we systematically mapped the repertoire of manifested RRBs during everyday life activities, with a special focus on motor stereotypies. The mapping was performed through systematic videotape analysis. RRBs were catalogued into four domains, i.e., motor, sensory, vocal, and intellective as well as according to their structure: simple (involving a single element among one of the domains) or complex (involving more than one element of the domains).

Notably, to characterize all the behaviors classified as repetitive, and not only motor or sensory ones, we expanded RRBs repertoire by adding behaviors that seemed to correspond to a need for sameness and a series of behaviors that involve complex motor and verbal sequences, as previously proposed by Militerni et al. [32].

Therefore, the aims of this study were (1) to analyze RRBs patterns through video recordings i.e., VICTOR project (VIdeo CaTaloguing OF Repetitive behaviors), and (2) to determine whether the number and quality of RRBs were related to ASD severity.

## 2. Materials and Methods

### 2.1. Participants

The study involved a group of 67 children and adolescents with a diagnosis of ASD according to the DSM-5 criteria [19] and a diagnosis confirmation based on the Autism Diagnostic Observation Scale—2nd edition criteria (ADOS-2 [33,34]). The ADOS-2 is a semi-structured, standardized measure of communication, social interaction, play/imagination, and restricted and/or repetitive behaviors [33]. It consists of 5 modules organized by language ability and chronological age, i.e., toddler module and modules 1 through 4. Five categories are administrated: Language and Communication, Mutual Social Interaction, Imagination and Creativity, Stereotyped Behaviors and Narrow Interests, Other Abnormal Behaviors. Scores are organized into two domains: Social Affect (ADOS SA), including Reciprocal Social Communication and Interaction, and Restricted and Repetitive Behavior (ADOS RRB). A calibrated score (ADOS CSS) is computed considering age and language level. The total score ranges from 0 to 10, with high scores indicating a more severe level of ASD-related symptoms.

All participants were residents and/or outpatients of a day care center for the rehabilitation and management of children and adolescents with ASD, multiple deficits, and intellectual disability of different severity. Intellectual disability was classified as mild, moderate, severe, and profound, according to the DSM-5 [19]. Trained expert clinicians in developmental psychology performed all clinical and diagnostic evaluations.

### 2.2. Procedures

Twenty expert healthcare professionals (all female, trained and graduated in special education, with long-standing professional experience) wearing a small body cam (dimension: 88.4 mm × 52.2 mm × 19.6 mm) placed on the thorax recorded specific RRBs in an ecological context during everyday life activities of the 67 subjects with ASD over three months with close follow-up. All healthcare professionals were previously trained to minimize their interaction with study participants while recording and recognizing the RRBs patterns.

Each participant was monitored carefully for five consecutive days a week. Each time the healthcare professional noticed the onset of RRBs, she started discretely video recording unbeknownst to the subject. In a subsequent recording event, the healthcare professional was asked to evaluate the possibility of interrupting each type of RRBs through two different extinction interventions: (1) verbal recall and/or proposal of an alternative activity or other appreciated stimulus: (2) physical guidance [35].

**Figure 1:**
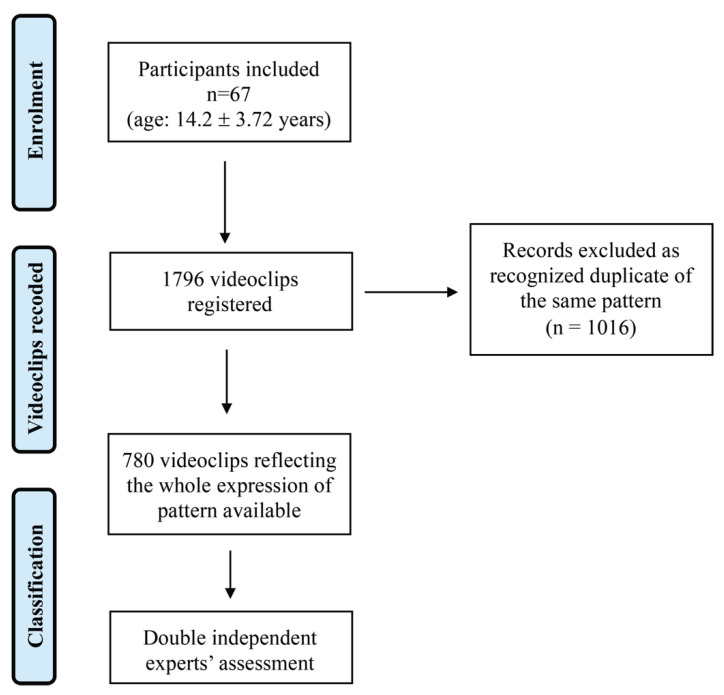
Flow diagram of video clips selection.

Approximately 1800 videos were obtained and later reviewed by an expert educator who selected and assembled them by discarding duplicates, enhancing and standardizing the quality and duration of the recordings as much as possible, thereby obtaining a video for each RRBs manifested by the subject. To this end, 780 videos concerning single RRBs were selected. The final version of the video library was saved in an appropriate server environment. Figure 1 shows the diagram flow of the video clips selection.

Then, the video library was used as the basis of the study by the team of expert reviewers. Then, the 780 videos (i.e., RRBs patterns) obtained were reviewed and analyzed by an expert team formed by two senior Child Neuropsychiatrists, a senior Developmental Psychologist, and the head of special educators involved in the study. The expert panel described and classified the detected RRB. The panel scored as RRB any apparently purposeless repetitive behaviors seen at least twice non-contiguously [31,36]. They subsequently compiled individual grids and loaded them into the database through a consensus strategy. Firstly, the panel of experts saw the video clips together. Then, each one assigned the category blinded to the others. The RRBs were classified as follows according to four domains, i.e., motor, sensory, vocal, and intellective, and their quality. The domains were selected based on previous literature [10,29,32]. Specifically, sensory RRBs were scored if the behavior results in a repetitive sensory input as described in previous studies [32,37].

The following categorization has been used to classify the components for each RRB domains:

- Simple Motor (M1): behavior consisting of one movement (e.g., finger tapping or hand waving, repetitive limb movements, body rocking, etc.);
- Complex Motor (M2): behavior consisting of repeated sequences of movements in several body districts (e.g., toe walking, jumping while walking or running, etc.);
- Simple Sensory (S1): involves a single sensorial aspect (e.g., touching an object or a surface, licking body parts or objects, etc.);
- Complex Sensory (S2): involves different sensory channels (e.g., grasping small items from the floor and put them in the mouth);
- Simple Vocal (V1): repeated simple vocalizations or “noises” (e.g., emitting grunt, raspberries, clearing throat, blowing, etc.);
- Complex Vocal (V2): repeating words phonemes, echolalia, coprolalia;
- Intellective: rigid, repetitive, stereotyped behaviors that express a need for routine, resistance to change, and a tendency to maintain environmental immutability. The Intellective domain was further divided between simple and complex behaviors as follows:
  - Simple Intellective (I1): simple rituals of rigid and repetitive behaviors that express a need for routine, resistance to change, e.g., crumble the food before eating it; always put the glass in the same place, keep the door of the cupboard open in the same way.
  - Complex Intellective (I2): complex ritual from the point of view of the reiterated behavioral sequence, e.g., trashing items while following the same path, line up different objects in the same order, etc.

Examples of RRBs are described in Supplementary Table S1. Once all the panelists scored the category, the evaluation was shared. In case of disagreement, an agreement was reached through discussion. Quality was defined as simple (involving a single element among one of the domains) and complex (involving more than one element of the domains) [32]. During the coding, the body parts and the sensory component involved were specified. For the body districts considered, please refer to the Result section. The modality of interruption was classified according to two categories: (1) verbal recall and/or proposal of an alternative activity or other appreciated stimulus; (2) physical guidance by the healthcare professional. Lastly, the prevalent subject state during the appearance of the RRBs was defined as quiet, agitated/excited, or both.

### 2.3. Statistical Analysis

Data are presented as number percentages or as means with SDs for nominal and continuous variables, respectively. Pearson correlation (r) analysis was used to investigate correlations as needed; frequency comparison and chi-square tests (χ^2^) were used for categorical variables. Mean comparisons were performed using paired T-tests for paired sample comparisons. Multiple comparisons were adjusted with Bonferroni corrections when appropriate. The level of significance was set at *p* < 0.05.

## 3. Results

Table 1 depicts the demographic characteristics of the participants involved in the study. Thirty-nine (58%) subjects had an ADOS-2 calibrated severity score (ADOS-2 CSS) showing high ASD severity. Forty-five subjects (67.2%) had a severe or profound intellectual disability, 19 subjects (28.4%) had a moderate intellectual disability, and three subjects (4.4%) had a mild intellectual disability. The RRBs spectrum ranged from one to 33 different types of patterns (mean = 11.6 ± 6.82; median = 10).

The most frequent pattern was represented by the combination of simple motor and sensorial components (i.e., M1 S1, accounting for 23% of the total number) followed by a simple motor (M1) and simple sensorial (S1) (9% and 8% respectively). The other 47 patterns with combinations from 1 to 4 components accounted for the remaining 60% with an asymmetric distribution of values. Table 2 shows in detail the frequency and percentage of each RRB pattern exhibited during the video recording.

Among the body parts involved during motor RRBs, the whole body and upper limb accounted for more than 74% (39% and 36.7%, respectively) (Table 3).

Within 569 out of 780 patterns containing at least one motor component, whole body and upper limb movements constituted the most common body parts involved (39% and 37% respectively) followed by mouth and hands (10% and 9.8% respectively). Considering the 531 out of 780 patterns containing at least one sensorial component, the most frequent sensory input involved was tactile (50%) followed by proprioceptive (34%) and acoustic (19.5%). Within the one-hundred twenty-seven RRBs with vocal components, there were 109 (85.2%) consisting of simple vocalizations and 18 (14.8%) consisting of phonemes or words.

Concerning the prevalent state, *n* = 183 (23%) of RRBs were shown during both quietness and agitated/excited state, while *n* = 566 (73%) and *n*= 31 (4%) were exhibited during quietness and agitated/excited state, respectively.

Among the 780 patterns, *n*= 417 (53.5%) required a physical intervention (e.g., physical guidance for interrupting the behaviors), while *n* = 363 (46.5%) needed a verbal interruption or a proposal of an alternative activity or other stimuli. However, a low and not significant correlation was found between RRBs pattern and interruption type (verbal or physical). Indeed, maximal correlation values (r) among fifty patterns and interruption modality ranged from −0.08 to +0.08.

Table 4 shows the comparison among subjects with a low number (≤5) and a high number (≥20) of RRBs, not accounting for their complexity features. No significant differences were found between the two groups for age and sex. Significant effects of ADOS-2 CSS (*p* = 0.044) and intellectual disability (*p* = 0.045) were found on the number of RRBs exhibited.

To investigate the relationship between RRBs quality (simple or complex) and autism severity according to the ADOS-2 CSS, a subject was considered to express complex RRBs when the proportion of complex over simple patterns exceeded 50%. Therefore, forty subjects (60%) were classified as expressing simple patterns and 27 (40%) participants were classified as expressing complex patterns. Table 5 shows the comparison between subjects with simple and complex patterns of RRBs. No significant differences were found between the two groups concerning age, sex, number of RRBs, and ADOS-2 CSS. A significant effect of intellectual disability (*p* = 0.044) was detected.

Table 6 depicts the correlation between the variables examined. Non-significant correlations were found between the total number of RRBs exhibited by the subject, age, and ADOS-2 scores for Social Affect subscale. Considering the ADOS-2 CSS as a dependent variable, the number of RRBs rather than their complexity features showed higher correlation values. The ADOS-2 RRBs subscale showed a higher correlation coefficient (r = 0.458) than ADOS-2 CSS (r = 0.347) concerning the number of subjects with a prevalent complex pattern of RRBs.

## 4. Discussion

Despite their strong diagnostic significance, RRBs remain a relatively gray area in autism research. The low interest for RRBs can be explained mainly by the difficulty in handling the high complexity of existing patterns of presentation.

Rather than classifying RRBs into two categories, we have proposed a classification based on a continuum construct according to the complexity of the behavior. Complexity dimensionality is based on the co-occurrence within the single behavior of motor, sensory, vocal, and cognitive components, and phenotypic complexity. The latter considers the number of body parts or sensory channels involved, the complexity of vocal behavior (sound/phonemes/words), or the sequence of the reiterated ritual.

### 4.1. Number of RRBs and Autism Severity

We found a significant direct relationship between the number of RRBs and ASD severity measured by the ADOS-2 CSS. This result was confirmed by the fact that subjects with a high number of RRBs (≥20) showed a significantly high ADOS-2 CSS compared to subjects with a low number of RRBs (≤5). This agrees with several studies that investigated the association between autism severity and RRBs. Most of them found that RRBs were more frequent as the severity of autism increased [31,38,39,40]. On the other hand, Bodfish et al. [41] reported a non-significant association between RRBs and the severity of ASD.

### 4.2. Pattern of RRB and Autism Severity

With our careful monitoring of subjects’ behavior, we found that a single subject might exhibit both complex and simple RRBs patterns, suggesting the need to classify at a subject level rather than at the RRBs level. Our pragmatic choice was to consider a participant as expressing complex RRB when the proportion of complex over simple patterns exceeds 50%. In our population, 40 subjects were classified accordingly as expressing prevalent simple pattern and 27 were classified as expressing prevalent complex patterns. Interestingly, when we compared the ADOS-2 CSS between subjects with prevalent simple and subjects with prevalent complex patterns of RRBs, no significant differences were found. This finding might be related to the approach we used for allocating subjects in one of two classes. The poor linear correlation among features of RRBs patterns and ADOS-2 CSS should prompt the use of a machine learning system approach.

Notably, also previous studies that assessed the relationship between RRBs and other clinical features in children with ASD found conflicting results. In a large sample of children and adolescents with ASD aged 4–18 years, Bishop et al. [42] reported an inverse correlation between ‘‘lower-level’’ RRBs and both non-verbal IQ and chronological age, whereas ‘‘higher-level’’ behaviors showed no relationship with IQ. Mirenda et al. [43] did not observe any significant relationship between RRBs and both non-verbal IQ and chronological age in a sample of 287 preschool-aged children with ASD. Joseph et al. [44] failed to find a significant relationship between RRBs and the non-verbal developmental quotient, chronological age, social communication, and sex in a sample of preschoolers with ASD. In a study of toddlers with ASD, Wolff et al. [45] observed that ‘‘higher-level’’ RRBs increased with chronological age. They reported that restricted behaviors were modestly negatively correlated with non-verbal developmental quotient at twelve months of age, suggesting that the relationship between RRBs and cognitive measures develops over time.

It is worth noting that the heterogeneity of participants with ASD across studies in terms of age, sex, cognitive function, and ASD severity may contribute to obtaining different and occasionally contradictory results and thus may have significantly interfered with a clear understanding of the RRBs profile in individuals with ASD [3]. No correlation was found between RRBs and interruption interventions, highlighting that the degree of RRBs complexity was not related to the type of interruption intervention.

### 4.3. Measuring RRBs in Individuals with ASD

Previously, Goldman and Greene [46] demonstrate the utility of video recording to assess repetitive behaviors in preschool children with ASD in a standardized play setting. Recently, a panel of experts reviewed questionnaires for assessing repetitive behaviors [30]. Among the most popular instruments to assess RRBs in individuals with ASD, there are The Yale–Brown Obsessive-Compulsive Scale [47,48], CY-BOCS [49], the Childhood Routines Inventory [50], the Repetitive Behavior Interview [51], the Repetitive Behavior Scale-Revised (RBS-R) [41], and the ‘‘Restricted interests and repetitive behaviors’’ section of Autism Diagnostic Interview-Revised (ADI-R) [52]. All of them are based on caregiver interviews or questionnaires and might suffer from psychometric limitations due to the Likert-based response type [53]. To our knowledge, this is the first study that directly investigates RRB and correlates their features with autism severity in an ecological environment. Thanks to this approach, a unique complex scenario appears with the discovery that certain subjects with autism can express more than 30 different patterns of RRBs.

Notably, the present work used a non-standard, real-world approach to observe RRBs in children and adolescence with ASD. Subjects were directly observed, and the RRBs were classified according to the recorded video clips. This approach mitigated the recall bias, which may be found on caregivers’ reports involving interviews or questionnaires. The video clips recorded through direct observation may also be a valid tool to investigate trajectories of specific types of stereotypies over time (e.g., longitudinal evaluation of RRBs [46]).

On the other hand, one of the limitations of the video recordings may be the use of bodycams that involved the health professionals training to interfere as little as possible with the daily activity of the participants.

### 4.4. Study Limitations and Strengths

This study has some limitations that should be acknowledged. Firstly, results should be interpreted in light of the cross-sectional design of the study and the sample size enrolled. A grouping by age to investigate RRB longitudinal trajectories was not performed due to the limited sample size that may lead to underpowered subgroup analyses. Video recordings may be biased due to experimenter–subject interaction. However, health professionals used small body cams and were trained to interfere as less as possible with the daily clinical routine of the participants. Self-injurious behaviors were not recorded. If a participant showed a harmful behavior (e.g., head banging, self-biting, etc.), the health professionals were trained to stop it and prevent injuries. The contexts and the specific activity (e.g., play, social interactions, mealtime, recreation) where the RRBs occurred were not reported. These limitations were mitigated by the detailed video recordings carried out during daily life activities in the facility. Moreover, participants were not exposed to new activities or new people.

To this end, this study deserves attention for its strengths: (1) A systematic mapping of the entire repertoire of manifested RRBs and their video recording in an ecological context (video analysis); (2) Classification of RRBs according to four behavioral domains (i.e., motor, sensory, vocal, and cognitive) and according to their phenotypic complexity, i.e., simple (involving a single motor, sensory, vocal, and cognitive domain) or complex (involving multiple domains); (3) An assessment of the relation between RRBs and sensory disturbances; (4) A proposal to define RRBs pattern complexity that could raise a methodological debate on this controversial topic.

### 4.5. Directions for Future Research

Progress in the understating of RRBs in ASD has been made [3]. Future research should address within-age differences in children and adolescents with ASD who exhibit low or high RRBs and different RRBs patterns. Studies should consider whether there are relationships between different RRBs and clinical/demographical characteristics in individuals with ASD. Additionally, research should explore the variety of RRBs across all domains concentrating specifically on each domain, e.g., motor RRBs. Studies should investigate the role of context and activities on the number and types of RRBs exhibited. Finally, it would be compelling to compare RRBs using different outcomes for ASD severity, such as the Childhood Autism Rating Scale and the Social Responsiveness Scale.

## 5. Conclusions

This study contributes to the understanding of RRBs in children and adolescents with ASD. Overall, our findings represent a first attempt to systematically classify the range of RRBs in a cohort of subjects with ASD closely followed by healthcare professionals in a natural environment. The emerging picture is a detailed description of the broad spectrum of RRBs at the individual level. Future research should use a bioinformatics approach such as a machine learning system or applying neural network architectures to classify and follow RRBs over time. The scientific community should shape the future research agenda for investigating RRBs in healthcare and in real-world settings. This may provide a better understanding of the pathophysiology, diagnosis, and treatment of RRBs. Future research should also investigate the correlation between IQ and RRBs. Moreover, studies about older populations with ASD need to be done to elucidate the natural course of RRBs in public and private settings.

## Figures and Tables

**Table 1:** Demographic and clinical characteristics of the ASD cohort.

Age Range—Years	7.1—24.8
Mean Age—years (SD)	14.2 (3.72)
Male *n*. (%)	58 (82%)
Female *n*. (%)	9 (18%)
Mean ADOS Total score (SA + RRB) (SD)	21.14 (5.34)
Mean ADOS CSS (SD)	7.93 (1.72)
ADOS CSS Range [Min, Max]	6 [4,10]
ADOS CSS—high severity *n*. subjects (%)	39 (58%)
ADOS CSS—moderate severity *n*. subjects (%)	26 (39%)
ADOS CSS—low severity *n*. subjects (%)	2 (3%)
Epilepsy co-occurrence	10 (15%)
Intellectual disability—mild *n*. subjects (%)	3 (4.48%)
Intellectual disability—moderate *n*. subjects (%)	19 (28.36%)
Intellectual disability—severe *n*. subjects (%)	44 (65.67%)
Intellectual disability—profound *n*. subjects (%)	1 (1.49%)

ADOS CSS, ADOS Calibrated Severity Score.

**Table 2:** Distribution of RRBs patterns of the sample.

RRBs Pattern	Frequency	RRBs Pattern	Frequency
M1 S1	23.08%	**V2**	0.51%
M1	8.97%	**M2 S1 I1**	0.51%
S1	7.95%	**S2 I1**	0.38%
**M2 S1**	7.95%	**M2 S2 I1**	0.38%
**I2**	4.62%	**I2 V1**	0.38%
**M2**	4.36%	**I1 V2**	0.26%
**M2 S2**	3.97%	**M2 S2 V2**	0.26%
**S2 M1**	3.97%	**V2 S1 I1**	0.26%
I1	3.59%	**I2 S1 M2**	0.26%
M1 S1 V1	3.08%	**M2 V1 I1**	0.26%
**S2**	2.44%	**S1 V2**	0.13%
**S2 M2 V1**	2.31%	**M1 V2**	0.13%
I1 M1	2.18%	**S2 M1 I1**	0.13%
S1 V1	2.18%	**M2 I1 V2**	0.13%
M1 V1	1.67%	**M2 V2**	0.13%
**M2 V1**	1.67%	**M1 S2 V2**	0.13%
**M2 S1 V1**	1.54%	**I2 S2 V2**	0.13%
S1 I1	1.41%	**I2 M2 S1 V1**	0.13%
**S2 M1 V1**	1.41%	**M2 S1 V2**	0.13%
**I1 M2**	1.41%	**M2 S2 V2 I1**	0.13%
**S2 V1**	1.28%	**I2 S2 M2**	0.13%
M1 S1 I1	1.15%	I1 M1 V1	0.13%
**I2 M2**	0.90%	M1 V1 S1 I1	0.13%
I2 S1	0.90%	**M2 S1 V1 I1**	0.13%
V1	0.64%	**M1 V1 I1 S2**	0.13%

Simple motor (M1), complex motor (M2), simple sensorial (S1), complex sensorial (S2), simple Intellective (I1), complex Intellective (I2), simple vocal (V1), complex vocal (V2). Bold: complex pattern.

**Table 3:** Body parts involved in motor stereotypies.

	N	Percentage
Body as a whole	222	39.0%
Upper limb	209	36.7%
Mouth	58	10.2%
Hands	57	10.0%
Head	37	6.5%
Eyes	22	3.9%
Face	17	3.0%
Ears	15	2.6%
Tongue	7	1.2%
Trunk	7	1.2%
Nose	6	1.1%
Feet	5	0.9%
Fingers	5	0.9%
Lower arms	3	0.5%
Teeth	2	0.4%

**Table 4:** Comparison among subjects with a low number (5 or less) and a high number (20 or more) of RRBs.

Variable	RRBs Low Number (*n* = 10)	RRBs High Number (*n* = 11)	Test	*p*-Value
Mean Age (SD)	14.5 (4.57)	13.8 (3.48)	T = −0.38	0.70
Male	8 (80%)	9 (82%)	χ^2^ = 0.0112	0.91
Female	2 (20%)	2 (18%)		
Mean ADOS CSS (SD)	7.2 (1.68)	8.7 (1.55)	t = −2.16	0.044 *
Intellectual Disability			χ^2^ = 6.20454	0.045 *
Moderate	5	1		
Severe	4	10		
Profound	1	-		

*n*, number of subjects; ADOS CSS, ADOS Calibrated Severity Score; * significant *p*-value.

**Table 5:** Comparison between 40 subjects with a prevalent simple pattern of RRBs and 27 subjects with a prevalent complex pattern of RRBs.

Variable	Simple Pattern Group (*n* = 40)	Complex Pattern Group (*n* = 27)	Test	*p*-Value
Mean Age (SD)	13.87 (3.88)	13.66 (3.47)	t = −0.22	0.81
Male	36 (90%)	22 (81%)	χ^2^ = 1.0059	0.32
Female	4 (10%)	5 (19%)		
Mean number of RRB (SD)	12.21 (7.82)	10.85 (5.43)	t = −0.79	0.43
Mean ADOS CSS (SD)	7.63 (1.88)	8.07 (1.26)	t = 1.06	0.29
Intellectual Disability			χ^2^ = 2.8778	0.044 *
Mild	3 (7.5%)	-		
Moderate	11 (27.5%)	8 (29.6%)		
Severe	25 (62.5%)	19 (70.4%)		
Profound	1 (2.5%)	-		

ADOS CSS, ADOS Calibrated Severity Score; * significant *p*-value.

**Table 6:** Correlation tests among principal variables.

Variables	N Stereotypies Per Subject	N Simple RRBs	N Complex RRBs	Age	ADOS SA	ADOS RRBs	ADOS CSS
N RRBs per subject	-						
N Simple RRBs	0.809 *	-					
N Complex RRBs	0.684 *	0.126	-				
Age	0.102	0.093	0.057	-			
ADOS SA	0.245	0.141	0.239	−0.025	-		
ADOS RRBs	0.425 *	0.210	0.458 *	0.148	0.500 *	-	
ADOS CSS	0.376 *	0.232	0.347 *	0.094	0.817 *	0.757 *	-

N, Number; ADOS SA, ADOS Social Affect subscale; ADOS RRB, ADOS Restricted Repetitive Behavior; ADOS CSS, ADOS Calibrated Severity Score. * Pearson (r) values with *p* < 0.05, Bonferroni corrected.

## Data Availability

The data that support the findings of the current study are available from the corresponding author [MG] upon reasonable request.

## Supplementary Materials

The following are available online at https://www.mdpi.com/article/10.3390/brainsci11060678/s1, Table S1: Example of restricted and repetitive behaviors across the four domains.
