# Supplementary material for: Patterns of Restricted and Repetitive Behaviors in Autism Spectrum Disorders: A Cross-Sectional Video Recording Study. Preliminary Report
Source: Brain Sci. 2021 May 21;11(6):678. doi: 10.3390/brainsci11060678 (PMC8224357; doi:10.3390/brainsci11060678)
Supplement: Supplementary file 1 [file brainsci-11-00678-s001.zip › brainsci-1165778-supplementary.pdf]

Supplementary Table S1. Example of restricted and repetitive behaviors across the four domains.

**M1 - Simple Motor: behavior consisting of one movement**
Finger tapping
Finger drumming
Hand waving
Repetitive limb movements: either hand or arm flapping/waving
Body rocking
Hair twisting
Regular, rhythmic, head movements: e.g., an up-and-down "yes" movement, a side-to-side "no" movement, head nodding, head banging, shoulder-to-shoulder movement
Mouth opening and closing
Facial grimacing
Eye blinking
**M2 - Complex Motor: behavior consisting of repeated sequences of movements in several body districts**
Toe walking, jumping while walking, or running
Arms flapping, and flexion and extension of the wrist while running
Arm swinging while running
Moving the upper limbs (waving) while either running or jumping
Skipping or hopping while walking
Teeth Chattering while leg shaking or kicking
Clapping hands while body rocking
Regular, rhythmic, head movements while either arm flapping or waving
**S1 - Simple Sensory: involves a single sensorial aspect**
Touching an object or a surface
Licking body parts or objects
Hand-sniffing
Hearing the sound produced by an object
Facing rubbing glass windows

Rubbing or touching the ear	
Ears closing/plugging with hands	
Biting hand	
**S2 - Complex Sensory: involves different sensory channels**	
Grasping small non-food items from the floor and putting them in the mouth	
Body rocking while biting the hand	
Touching and smelling the hairs	
Touching and smelling the hand	
Putting the hand in the mouth while licking and smelling it	
Looking and listening to a sound object	
Ears closing/plugging with hands while touching lips	
**V1 - Simple Vocal: repeated simple vocalizations or "noises," acontextual sounds**	
Vocal remitting grunt	
Raspberries made by bilabial trill produced by lips	
Babbling	
Clearing throat	
Blowing	
**V2 - Complex Vocal: repeating words phonemes, acontextual or nonfunctional speech/words**	
Echolalia	
Coprolalia	
Repeating phrases unrelated to the present situation	
**Intellective: rigid, repetitive, stereotyped behaviors that express a need for routine, resistance to change, and a tendency to maintain environmental immutability. The Intellective domain was further divided between simple (I1) and complex behaviors (I2)**	
I1 - Simple Intellective: simple rituals of rigid and repetitive behaviors that express a need for routine and resistance to change	
Arranging objects in a specific order or pattern	
Crumbling the food before eating it	
Arranging the glass in the same place	

Keeping the door of the cupboard open in the same position
Walking up the stairs two steps at the time
Pencil sharpening
Picking at finger nails and cuticles
Refusing to walk on specific ground (e.g., grass)
I2 - Complex Intellective: complex ritual from the point of view of the reiterated behavioral sequence
Trashing items while following the same route
Walking, turning around, and stopping.
Pacing and walking in circles
Coughing while either hand or arm flapping/waving
Keeping the cabinet door open and moving the door slightly to hear the noise
Walking in circles before sitting.
Insistence of walking on the same route before entering a room
Insistence on turning in a circle times before entering a room
Aligning shoes before wearing shoes
